# Supplementary material for: Identification and Characterization of Novel Sources of Resistance to Rust Caused by Uromyces pisi in Pisum spp
Source: Plants (Basel). 2022 Aug 31;11(17):2268. doi: 10.3390/plants11172268 (PMC9460634; doi:10.3390/plants11172268)
Supplement: Supplementary file 1 [file plants-11-02268-s001.zip › plants-1870989-supplementary.pdf]

Supplementary Table S1. Accession list including bank code, bank origin, taxonomy, common name, germplasm origin and material type.

Bank code	Bank origin	Taxonomy	Common name	Germplasm origin	Material type
PI 109865	USDA	*P. sativum* L.	ARVEJAS AMARILLAS	Venezuela	Landraces
PI 117910	USDA	*P. sativum* L.	ERVILHA ANA	Brazil	Cultivar
PI 140297	USDA	*P. sativum* subsp. *sativum*	No. 6192	Iran	Landraces
PI 142442	USDA	*P. sativum* L.	ALBERJON	Peru	Cultivar
PI 142774	USDA	*P. sativum* L.	G 1704	Mexico	Landraces
PI 142776	USDA	*P. sativum* L.	G 1705	Mexico	Landraces
PI 142776	USDA	*P. sativum* L.	G 1705	Mexico	Landraces
PI 143483	USDA	*P. sativum* L.	No. 7351	Azerbaijan	Landraces
PI 143484	USDA	*P. sativum* L.	CPI 135298	Azerbaijan	Landraces
PI 143486	USDA	*P. sativum* L.	No. 7790	Iran	Landraces
PI 153351	USDA	*P. sativum* L.	ARVEJAS VERDES	Ecuador	Landraces
PI 162568	USDA	*P. sativum* L.	ORGULLO DEL MERCADA	Argentina	Cultivar
PI 162692	USDA	*P. sativum* L.	CUARENTONA	Argentina	Cultivar
PI 162693	USDA	*P. sativum* L.	OJO NEGRO	Argentina	Cultivar
PI 162693	USDA	*P. sativum* L.	OJO NEGRO	Argentina	Cultivar
PI 162910	USDA	*P. sativum* L.	L.P. NO. 7	Paraguay	Landraces
PI 164568	USDA	*P. sativum* L.	Patani	India	Landraces
PI 166082	USDA	*P. sativum* subsp. *sativum*	Matar	India	Landraces
PI 195405	USDA	*P. sativum* L.	QUEZALTENANGO	Guatemala	Landraces
PI 203065	USDA	*P. sativum* L.	G 6821	Finland	Landraces
PI 204305	USDA	*P. sativum* L.	COLLEGIAN	Australia	Cultivar
PI 204667	USDA	*P. sativum* subsp. *sativum* var. *sativum*	STIJFSTRO	Netherland	Cultivar
PI 220175	USDA	*P. sativum* L.	No. 150	Afganistan	Landraces
PI 220673	USDA	*P. sativum* L.	Moshong	Afganistan	Landraces
PI 222069	USDA	*P. sativum* L.	Moshong	Afganistan	wild
PI 234262	USDA	*P. sativum* L.	Carlon	USA	Cultivar
PI 254625	USDA	*P. sativum* L.	KELLERVA	Finland	Landraces
PI 254626	USDA	*P. sativum* L.	LIMA	Australia	Cultivar
PI 261678	USDA	*P. sativum* L.	Col. No. D-237	Netherland	Landraces
PI 262189	USDA	*P. sativum* L.	BIG PEA	Costa Rica	Landraces
PI 266069	USDA	*P. sativum* L.	LINE NO. 110	Sweden	breeding lines
PI 269760	USDA	*P. sativum* subsp. *sativum* var. *arvense* (L.) Poir.	G 16701	UK	breeding lines
PI 269763	USDA	*P. sativum* subsp. *jomardii* (Schrank) Kosterin	Aa86	UK	Landraces
PI 269786	USDA	*P. sativum* L.	Aa96	UK	Landraces
PI 272143	USDA	*P. sativum* subsp. *thebaicum*	THEBAICUM RUBY	Germany	Landraces
PI 272151	USDA	*P. sativum* L.	UNIFLORUM	Germany	Landraces
PI 272153	USDA	*P. sativum* L.	HIEMALE	Greece	Landraces
PI 272156	USDA	*P. sativum* L.	HIEMALE	Greece	Landraces
PI 280621	USDA	*P. sativum* L.	AMPLISSIMO SPARTANEC	Rusia	Landraces
PI 280623	USDA	*P. sativum* L.	AMPLISSIMO PULAVSKIJ	Poland	Landraces
PI 306592	USDA	*P. sativum* L.	G 19029	Hungary	Landraces
PI 312136	USDA	*P. sativum* L.	ALBERGA	Guatemala	Landraces
PI 319373	USDA	*P. sativum* L.	CHICHARO SERRANO	Mexico	Landraces
PI 326194	USDA	*P. sativum* L.	Col. No. 22340	Mexico	Landraces
PI 343326	USDA	*P. sativum* L.	G 18456	USA	Landraces
PI 343329	USDA	*P. sativum* L.	G 18459	USA	Landraces
PI 343935	USDA	*P. sativum* L.	6922	Ethiopia	Landraces
PI 343962	USDA	*P. sativum* L.	22662	Turkey	wild
PI 343965	USDA	*P. sativum* L.	22706	Turkey	Landraces
PI 343965	USDA	*P. sativum* L.	22706	Turkey	Landraces
PI 343969	USDA	*P. sativum* subsp. *sativum* var. *sativum*	ARAKA	Turkey	Landraces
PI 343981	USDA	*P. sativum* subsp. *sativum* var. *sativum*	22654	Turkey	Landraces
PI 343984	USDA	*P. sativum* subsp. *sativum*	22712	Turkey	Landraces
PI 343993	USDA	*P. sativum* subsp. *sativum* var. *arvense* (L.) Poir.	22652	Turkey	Landraces
PI 347282	USDA	*P. sativum* L.	PLP 11	India	Landraces
PI 347316	USDA	*P. sativum* L.	PLP 68	India	Landraces
PI 347317	USDA	*P. sativum* L.	PLP 71	India	Landraces
PI 347319	USDA	*P. sativum* L.	PLP 73	India	Landraces
PI 347321	USDA	*P. sativum* L.	PLP 88	India	Landraces
PI 347323	USDA	*P. sativum* L.	PLP 89	India	Landraces
PI 347326	USDA	*P. sativum* L.	PLP 93	India	Landraces
PI 347328	USDA	*P. sativum* L.	PLP 99	India	Landraces
PI 347330	USDA	*P. sativum* L.	PLP 102	India	Landraces
PI 347332	USDA	*P. sativum* L.	PLP 104	India	Landraces
PI 347333	USDA	*P. sativum* L.	PLP 105	India	Landraces
PI 347334	USDA	*P. sativum* L.	PLP 109	India	Landraces
PI 347335	USDA	*P. sativum* L.	PLP 113	India	Landraces
PI 347336	USDA	*P. sativum* L.	PLP 118	India	Landraces
PI 347338	USDA	*P. sativum* L.	PLP 126	India	Landraces
PI 347342	USDA	*P. sativum* L.	PLP 154	India	Landraces
PI 347343	USDA	*P. sativum* L.	PLP 156	India	Landraces
PI 347347	USDA	*P. sativum* L.	PLP 173	India	Landraces
PI 347348	USDA	*P. sativum* L.	PLP 182	India	Landraces
PI 347356	USDA	*P. sativum* L.	PLP 218	India	Landraces
PI 347357	USDA	*P. sativum* L.	PLP 219	India	Landraces

PI 347359	USDA	*P. sativum* L.	PLP 222	India	Landraces
PI 347366	USDA	*P. sativum* L.	PLP 266	India	Landraces
PI 347367	USDA	*P. sativum* L.	PLP 268	India	Landraces
PI 347370	USDA	*P. sativum* L.	PLP 278	India	Landraces
PI 347372	USDA	*P. sativum* L.	PLP 297	India	Landraces
PI 347373	USDA	*P. sativum* L.	PLP 301	India	Landraces
PI 347374	USDA	*P. sativum* L.	PLP 303	India	Landraces
PI 347375	USDA	*P. sativum* L.	PLP 304	India	Landraces
PI 347383	USDA	*P. sativum* L.	PLP 316	India	Landraces
PI 347385	USDA	*P. sativum* L.	PLP 320	India	Landraces
PI 347388	USDA	*P. sativum* L.	PLP 330	India	Landraces
PI 347389	USDA	*P. sativum* L.	PLP 332	India	Landraces
PI 347401	USDA	*P. sativum* L.	PLP 363	India	Landraces
PI 347471	USDA	*P. sativum* L.	PLP 450	India	Landraces
PI 358642	USDA	*P. sativum* subsp. *sativum* var. *arvense* (L.) Poir.	22793	Ethiopia	Landraces
PI 379612	USDA	*P. sativum* L.	WEIBULL 700	Sweden	Cultivar
PI 385981	USDA	*P. sativum* L.	ONWARD	UK	Cultivar
PI 399129	USDA	*P. sativum* L.	FLORIDA	Germany	Cultivar
PI 494079	USDA	*P. sativum* L.	G 27917	Chile	Landraces
PI 560065	USDA	*P. fulvum* Sm.	CPI 134669	Israel	wild
PI 560067	USDA	*P. fulvum* Sm.	CPI 134471	Israel	wild
PI 595933	USDA	*P. fulvum* Sm.	ATC 113	Australia	wild
PI 595945	USDA	*P. fulvum* Sm.	CPI 53306	Jordan	wild
PI 595947	USDA	*P. fulvum* Sm.	VIR 2523	Israel	wild
JI 85	JIC UK	*P. sativum* L.	P.SATIVUM-AFGHANISTAN	Afganistan	Landraces
JI 156	JIC UK	*P. sativum* L.	P.SATIVUM-USSR	Sudan	Landraces
JI 156	JIC UK	*P. sativum* L.	P.SATIVUM-USSR	Sudan	Landraces
JI 262	JIC UK	*P. sativum* subsp. *elatius* var. *elatius* (M. Bieb.) Alef.	P. ELATIUS	Turkey	wild
JI 263	JIC UK	*P. sativum* subsp. *sativum* var. *arvense* (L.) Poir.	P.SATIVUM-BALKANS	Greece	wild
JI 228	JIC UK	*P. sativum* L.	P.SATIVUM-BOLIVIA	Bolivia	Landraces
JI 209	JIC UK	*P. sativum* subsp. *sativum* var. *arvense* (L.) Poir.	P.SATIVUM ARVENSE	India	Landraces
JI 209	JIC UK	*P. sativum* subsp. *sativum* var. *arvense* (L.) Poir.	P.SATIVUM ARVENSE	India	Landraces
JI 207	JIC UK	*P. sativum* subsp. *sativum* var. *sativum*	P.SATIVUM CHORESMICUM	UZBEKISTAN	Landraces
JI 224	JIC UK	*P. fulvum* Sm.	P. FULVUM	Israel	wild
JI 196	JIC UK	*P. sativum* subsp. *transcaucasicum* Govorov	P.SATIVUM-GEORGIA	Georgia	Landraces
JI 190	JIC UK	*P. sativum* L.	wiraig	Sudan	Landraces
JI 189	JIC UK	*P. sativum* L.	wiraig	Sudan	Landraces
JI 185	JIC UK	*P. sativum* L.	wiraig	Sudan	Landraces
JI 267	JIC UK	*P. sativum* L.	P.SATIVUM-GREECE	Greece	wild
JI 268	JIC UK	*P. sativum* L.	P.SATIVUM-CRETE	Crete	wild
JI 275	JIC UK	*P. sativum* L.	P.SATIVUM-CRETE	Crete	wild
JI 280	JIC UK	*P. sativum* L.	P.SATIVUM-ALBANIA	Albania	wild
JI 288	JIC UK	*P. sativum* L.	P.SATIVUM-GREECE	Greece	wild
JI 502	JIC UK	*P. sativum* L.	Rondo	Netherland	Cultivar
JI 701	JIC UK	*P. sativum* L.	P.SATIVUM-ITALY	Italy	Landraces
JI 1030	JIC UK	*P. sativum* L.	P.SATIVUM-IRAN	Iran	Landraces
JI 1057	JIC UK	*P. sativum* L.	ANTIOQUIA I CHILENA	Colombia	Landraces
JI 1089	JIC UK	*P. sativum* subsp. *sativum* var. *arvense* (L.) Poir.	P.elatius	Turkey	Landraces
JI 1107	JIC UK	*P. sativum* L.	keerau pea	Nepal	Landraces
JI 1213	JIC UK	*P. sativum* L.	erylis	France	Cultivar
JI 1345	JIC UK	*P. sativum* L.	P.SATIVUM-MONGOLIA	Mongolia	Landraces
JI 1346	JIC UK	*P. sativum* L.	P.SATIVUM-MONGOLIA	Mongolia	Landraces
JI 2263	JIC UK	*P. sativum* L.	WILD TUNESIAN	Germany	Landraces
JI 2265	JIC UK	*P. sativum* L.	P.SATIVUM VAR. HIEMALE	Albania	Landraces
JI 2356	JIC UK	*P. sativum* L.	P.SATIVUM-NEPAL	Nepal	Landraces
JI 2385	JIC UK	*P. abyssinicum* A. Braun	PISUM SP.-YEMEN	Yemen	Landraces
JI 2387	JIC UK	*P. sativum* L.	P.SATIVUM-Ethiopia	Ethiopia	Landraces
JI 2545	JIC UK	*P. sativum* L.	P. SATIVUM-PAKISTAN	Pakistan	Landraces
BGE001004	CRF INIA	*P. sativum* subsp. *sativum*	Garvanzo enano	Spain	Landraces
BGE001034	CRF INIA	*P. sativum* subsp. *sativum* var. *sativum*	Pesol	Spain	Landraces
BGE001121	CRF INIA	*P. sativum* subsp. *sativum* var. *sativum*	Negrer	Spain	Landraces
BGE001121	CRF INIA	*P. sativum* subsp. *sativum* var. *sativum*	Negrer	Spain	Landraces
BGE001662	CRF INIA	*P. sativum* subsp. *sativum*	Chicharo	Spain	Landraces
BGE002168	CRF INIA	*P. sativum* subsp. *sativum*	Tito	Spain	Landraces
BGE002168	CRF INIA	*P. sativum* subsp. *sativum*	Tito	Spain	Landraces
BGE003315	CRF INIA	*P. sativum* subsp. *sativum*	Tirabeque	Spain	Landraces
BGE004710	CRF INIA	*P. sativum* subsp. *sativum*	Ervilha	Portugal	Landraces
BGE004713	CRF INIA	*P. sativum* subsp. *sativum*	Ervilha	Portugal	Landraces
BGE004958	CRF INIA	*P. sativum* subsp. *sativum*	Ervilhoto	Portugal	Landraces
BGE006125	CRF INIA	*P. sativum* subsp. *sativum*	Grizeu farroba	Portugal	Landraces
BGE006126	CRF INIA	*P. sativum* subsp. *sativum* var. *sativum*	Ervilha	Portugal	Landraces
BGE019594	CRF INIA	*P. sativum* subsp. *sativum*	Arveja	Spain	Landraces
BGE022159	CRF INIA	*P. sativum* subsp. *sativum* var. *arvense* (L.) Poir.	Bisalto	Spain	Landraces
BGE020326	CRF INIA	*P. sativum* subsp. *sativum* var. *arvense* (L.) Poir.	Bisalto del terreno	Spain	Landraces
BGE023256	CRF INIA	*P. sativum* subsp. *sativum*	Guisante	Spain	Landraces
BGE025263	CRF INIA	*P. sativum* subsp. *sativum* var. *sativum*	Guisante verde	Spain	Landraces
BGE025267	CRF INIA	*P. sativum* subsp. *sativum*	Mangano;Presol;Guisante claro	Spain	Landraces
BGE025270	CRF INIA	*P. sativum* subsp. *sativum*	Guisante negro	Spain	Landraces
BGE026428	CRF INIA	*P. sativum* subsp. *sativum* var. *sativum*	Guisante rastrero	Spain	Landraces
BGE026429	CRF INIA	*P. sativum* subsp. *sativum*	Arvilla	Spain	Landraces
CGN16690	CGN	*P. sativum* L.		Italy	Landraces

CGN03277	CGN	*P. sativum* L.	NPE 378	Pakistan	Landraces
CGN13253	CGN	*P. sativum* L.	P.SATIVUM-Ethiopia	Ethiopia	Landraces
CGN16640	CGN	*P. sativum* L.	Khadraa	Sudan	Landraces
CGN16562	CGN	*P. sativum* L.	JI 1543	Mongolia	wild
CGN16571	CGN	*P. sativum* subsp. *jomardii* (Schrank) Kosterin	P. jomardii	Egypt	wild
CGN16581	CGN	*P. sativum* L.	JI 93	Afganistan	Landraces
CGN16639	CGN	*P. sativum* L.	JI 171	Ethiopia	Landraces
CGN16679	CGN	*P. sativum* subsp. *cinereum* Govorov	JI 204	Russia	wild
CGN16582	CGN	*P. sativum* L.	Keerau pea	Nepal	Landraces
CGN16684	CGN	*P. sativum* L.		Greece	Landraces
CGN16646	CGN	*P. sativum* L.		Mongolia	Landraces
CGN16636	CGN	*P. abyssinicum* A. Braun	P. abyssinicum	Ethiopia	Landraces
CGN03328	CGN	*P. sativum* L.	NPE 1210.362A	Pakistan	Landraces
CGN03170	CGN	*P. sativum* L.	Turkey-19	Irak	Landraces
CGN03190	CGN	*P. sativum* L.	Kulur	Turkey	Landraces
CGN03245	CGN	*P. sativum* L.	Ethiopia-32	Ethiopia	Landraces
CGN03165	CGN	*P. sativum* L.	Turkey-16	Turkey	Landraces
CGN03289	CGN	*P. sativum* L.	NPE 1175.346	Pakistan	Landraces
CGN03171	CGN	*P. sativum* L.	Selection 266/1	Turkey	breeding lines
CGN03290	CGN	*P. sativum* L.	NPE 1180.392	Pakistan	Landraces
CGN03305	CGN	*P. sativum* L.	NPE 1169.248	Pakistan	Landraces
CGN02921	CGN	*P. sativum* subsp. *sativum* var. *sativum*	Semi Nano Ideal	Italy	Cultivar
CGN03003	CGN	*P. sativum* subsp. *sativum* var. *sativum*	Petit Provencal	France	Cultivar
CGN03273	CGN	*P. sativum* L.	950 3e	Peru	breeding lines
CGN03229	CGN	*P. sativum* L.	Ethiopia-31	Ethiopia	Landraces
PI 413686	USDA	*P. sativum* L.	FELICITAS	Hungary	Cultivar
PI 477371	USDA	*P. sativum* L.	ROSAKRONE	Denmark	Cultivar
PI 307666	USDA	*P. sativum* L.	VERJA	Costa Rica	Landraces
PI 307666	USDA	*P. sativum* L.	VERJA	Costa Rica	Landraces
PI 324693	USDA	*P. sativum* L.	ABESINIJAS	Hungary	Cultivar
PI 324705	USDA	*P. sativum* L.	No. 830	France	Unknown
PI 355905	USDA	*P. sativum* subsp. *sativum* var. *sativum*	KAIRYO AOTENASHI	Japan	Cultivar
PI 241593	USDA	*P. sativum* L.	G 6571	Taiwan	Unknown
PI 273207	USDA	*P. sativum* subsp. *elatius* (M. Bieb.) Asch. & Graebn.	9006/60	Bulgaria	Landraces
PI 266070	USDA	*P. sativum* subsp. *sativum* var. *sativum*	LINE NO. 930	Sweden	breeding lines
PI 198074	USDA	*P. sativum* L.	GORSDAGSART III	Sweden	Landraces
PI 357292	USDA	*P. sativum* subsp. *sativum* var. *sativum*	KIFLICA	North Macedonia	Cultivar
PI 357293	USDA	*P. sativum* subsp. *sativum* var. *sativum*	DEBARSKI	North Macedonia	Cultivar
PI 249645	USDA	*P. sativum* L.	B.R. 178	India	Landraces
PI 357048	USDA	*P. sativum* subsp. *elatius* (M. Bieb.) Asch. & Graebn.	PLP 514	India	wild
PI 357289	USDA	*P. sativum* subsp. *sativum* var. *sativum*	RAN	North Macedonia	Cultivar
PI 253968	USDA	*P. sativum* subsp. *elatius* (M. Bieb.) Asch. & Graebn.	Col. No. K1722	Afganistan	Landraces
PI 103058	USDA	*P. sativum* L.	No. 10	China	Cultivar
PI 180329	USDA	*P. sativum* L.	Watana	India	Landraces
PI 184131	USDA	*P. sativum* subsp. *sativum* var. *sativum*	No. 310	Serbia	Landraces
PI 124478	USDA	*P. sativum* L.	Matar	Pakistan	Landraces
PI 124479	USDA	*P. sativum* L.	Matar	Pakistan	Landraces
PI 124479	USDA	*P. sativum* L.	Matar	Pakistan	Landraces
JI 2480	JIC	*P. sativum* L.	CGN 3352	Peru	breeding lines
JI 1951	JIC	*P. sativum* L.	P.SATIVUM-CHINA	China	Cultivar
JI 2302	JIC	*P. sativum* subsp. *sativum* var. *sativum*	B76-197 (STRATAGEM)	Sweden	breeding lines
JI 1566	JIC	*P. sativum* L.	Almota	USA	Cultivar
PI 608038	USDA	*P. sativum* L.	74SN5	USA	Cultivar
PI 613100	USDA	*P. sativum* L.	MINI	USA	Cultivar
Atc-4235-53	Commercial	*P. sativum* L.	Atc-4235-53	Australia	breeding lines
Boreen	Commercial	*P. sativum* L.	Boreen	Australia	Cultivar
Danclale	Commercial	*P. sativum* L.	Danclale	Australia	Cultivar
Kagpa	Commercial	*P. sativum* L.	Kagpa	Australia	Cultivar
M5	Commercial	*P. sativum* L.	M5	Australia	Cultivar
Pinochio	Commercial	*P. sativum* L.	Pinochio	Denmark	Cultivar
B 99-114	Commercial	*P. sativum* L.	B 99-114	Czech Republic	breeding lines
AGT 205,21	Commercial	*P. sativum* L.	AGT 205,21	Czech Republic	breeding lines
Morris	Commercial	*P. sativum* L.	Morris	Czech Republic	Cultivar
JI 1210	JIC	*P. sativum* L.	erygel	France	Cultivar
JI 1412	JIC	*P. sativum* L.	Marlin	USA	Cultivar
JI 1559	JIC	*P. sativum* L.	Mexique 4	Mexico	Cultivar
JI 1747	JIC	*P. sativum* L.	Almires	Germany	Cultivar
JI 1760	JIC	*P. sativum* subsp. *sativum* var. *sativum*	Consort-af	UK	Cultivar
JI 210	JIC	*P. sativum* L.	Lucknow Boniya	India	Cultivar
JI 252	JIC	*P. sativum* L.	P.SATIVUM-Ethiopia	Ethiopia	Landraces
JI 82	JIC	*P. sativum* L.	P.SATIVUM-AFGHANISTAN	Afganistan	Landraces
Messire	IAS	*P. sativum* subsp. *sativum* var. *sativum*	Messire	France	Cultivar
Radley	IAS	*P. sativum* subsp. *sativum* var. *sativum*	Radley	UK	Cultivar
Ballet	IAS	*P. sativum* subsp. *sativum* var. *sativum*	Ballet	UK	Cultivar
W6 17515	USDA	*P. sativum* subsp. *sativum* var. *sativum*	LITTLE MARVEL	USA	Cultivar
W6 17516	USDA	*P. sativum* subsp. *sativum* var. *sativum*	DARK SKIN PERFECTION	USA	Cultivar
W6 17517	USDA	*P. sativum* subsp. *sativum* var. *sativum*	NEW ERA	USA	Cultivar
W6 17518	USDA	*P. sativum* subsp. *sativum* var. *sativum*	NEW SEASON	USA	Cultivar
W6 17520	USDA	*P. sativum* subsp. *sativum* var. *sativum*	WSU 28	USA	Cultivar
KEBBY	Commercial	*P. sativum* subsp. *sativum* var. *sativum*	Kebby	UK	Cultivar
POLAR	Commercial	*P. sativum* subsp. *sativum* var. *sativum*	Polar	Spain	Cultivar

W6 17519	USDA	*P. sativum* subsp. *sativum* var. *sativum*	WSU 23	Unknow	Cultivar
W6 17521	USDA	*P. sativum* subsp. *sativum* var. *sativum*	WSU 31	Unknow	Cultivar
BGE023667	INIA	*P. sativum* subsp. *sativum*	Guisante	Spain	Landraces
BGE025727	INIA	*P. sativum* subsp. *sativum*	Guisante	Spain	Landraces
PI 358608	USDA-EEUU	*P. sativum* subsp. *sativum* var. *arvense* (L.) Poir.	22770B	Ethiopia	Landraces
PI 358609	USDA-EEUU	*P. abyssinicum* A. Braun	WAT	Ethiopia	wild
PI 173055	USDA-EEUU	*P. sativum* subsp. *elatius* var. *elatius* (M. Bieb.) Alef.	HATUN BAKLERI	Turkey	Landraces
PI 120617	USDA-EEUU	*P. sativum* subsp. *elatius* var. *elatius* (M. Bieb.) Alef.	No. 738	Turkey	Landraces
PI 273209	USDA-EEUU	*P. sativum* subsp. *elatius* var. *elatius* (M. Bieb.) Alef.	9009/60	Russia	Landraces
PI 344003	USDA-EEUU	*P. sativum* subsp. *elatius* var. *elatius* (M. Bieb.) Alef.	22703	Turkey	wild
PI 344005	USDA-EEUU	*P. sativum* subsp. *elatius* var. *elatius* (M. Bieb.) Alef.	22611	Greece	wild
PI 344006	USDA-EEUU	*P. sativum* subsp. *elatius* var. *elatius* (M. Bieb.) Alef.	22618	Greece	wild
PI 343976	USDA-EEUU	*P. sativum* subsp. *elatius* var. *elatius* (M. Bieb.) Alef.	22716	Turkey	wild
PI 505059	USDA-EEUU	*P. sativum* subsp. *elatius* var. *elatius* (M. Bieb.) Alef.	ILCA 5076	Sudan	Landraces
PI 344010	USDA-EEUU	*P. sativum* subsp. *elatius* var. *elatius* (M. Bieb.) Alef.	22732	Greece	wild
PI 344011	USDA-EEUU	*P. sativum* subsp. *elatius* var. *elatius* (M. Bieb.) Alef.	22733	Greece	wild
PI 344013	USDA-EEUU	*P. sativum* subsp. *elatius* var. *elatius* (M. Bieb.) Alef.	22735	Greece	wild
PI 116056	USDA-EEUU	*P. sativum* subsp. *sativum*	Matar	India	Landraces
PI 505127	USDA-EEUU	*P. sativum* subsp. *sativum*	ILCA 5094	Albania	Landraces
PI 242027	USDA-EEUU	*P. sativum* subsp. *jomardii* (Schrank) Kosterin	G 11764	Denmark	Unknown
PI 269762	USDA-EEUU	*P. sativum* subsp. *jomardii* (Schrank) Kosterin	Aa38	UK	Landraces
PI 343987	USDA-EEUU	*P. sativum* subsp. *sativum* var. *sativum*	22718	Turkey	Landraces
PI 505080	USDA-EEUU	*P. sativum* subsp. *sativum*	ILCA 5039	Cyprus	Unknown
PI 505111	USDA-EEUU	*P. sativum* subsp. *sativum*	ILCA 5075	Syria	Landraces
PI 268480	USDA-EEUU	*P. sativum* subsp. *elatius* var. *pumilio* Meikle	Col. No. 317	Afganistan	Landraces
JI 45	JIC	*P. sativum* subsp. *transcaucasicum* Govorov	P.TRANSCAUCASICUM	Georgia	wild
JI 198	JIC	*P. sativum* subsp. *elatius* var. *elatius* (M. Bieb.) Alef.	P. ELATIUS	Israel	wild
JI 199	JIC	*P. sativum* subsp. *elatius* var. *elatius* (M. Bieb.) Alef.	P. ELATIUS	Israel	wild
JI 225	JIC	*P. abyssinicum* A. Braun	P. ABYSSINICUM	Ethiopia	Landraces
JI 227	JIC	*P. abyssinicum* A. Braun	P. ABYSSINICUM	Ethiopia	Landraces
JI 241	JIC	*P. sativum* subsp. *elatius* var. *pumilio* Meikle	P. HUMILE	Israel	wild
JI 254	JIC	*P. sativum* subsp. *elatius* var. *elatius* (M. Bieb.) Alef.	P. ELATIUS	Ethiopia	wild
JI 804	JIC	*P. sativum* subsp. *sativum* var. *sativum*	P.TIBETANICUM	Unknow	Landraces
JI 1398	JIC	*P. sativum* L.	P.SATIVUM	China	Landraces
JI 1428	JIC	*P. sativum* subsp. *sativum* var. *sativum*	P.TIBETANICUM	Tibet	wild
JI 1854	JIC	*P. sativum* subsp. *elatius* var. *pumilio* Meikle	P. HUMILE	Israel	Landraces
JI 2116	JIC	*P. sativum* subsp. *sativum* var. *sativum*	P.SPECIOSUM	Spain	Landraces
JI 2202	JIC	*P. abyssinicum* A. Braun	P. ABYSSINICUM	Yemen	Landraces
PIS 1318/91	IPK	*P. sativum* subsp. *elatius* (M. Bieb.) Asch. & Graebn.		Unknow	Unknown
CGN10205	CGN	*P. sativum* subsp. *elatius* var. *elatius* (M. Bieb.) Alef.	1140175	Turkey	Landraces
CGN10206	CGN	*P. sativum* subsp. *elatius* var. *elatius* (M. Bieb.) Alef.	1145176	Unknow	breeding lines
CGN10193	CNG	*P. sativum* subsp. *sativum* var. *arvense* (L.) Poir.		Unknow	Unknown
IFPI 3365	ICARDA-Siria	*P. sativum* subsp. *elatius* var. *elatius* (M. Bieb.) Alef.	IG 52524	Turkey	wild
IFPI 3370	ICARDA-Siria	*P. sativum* subsp. *elatius* var. *elatius* (M. Bieb.) Alef.	IG 52529	Turkey	wild
IFPI 387	ICARDA-Siria	*P. sativum* subsp. *thebaicum*	IG 49546	USSR	wild
IFPI 436	ICARDA-Siria	*P. sativum* subsp. *jomardii* (Schrank) Kosterin	IG 49595	Egypt	Landraces
IFPI 2348	ICARDA-Siria	*P. sativum* subsp. *sativum* var. *arvense* (L.) Poir.	IG 51507	Ethiopia	Landraces
IFPI 2350	ICARDA-Siria	*P. sativum* subsp. *sativum* var. *arvense* (L.) Poir.	IG 51509	Ethiopia	Landraces
IFPI 2351	ICARDA-Siria	*P. sativum* subsp. *sativum* var. *arvense* (L.) Poir.	IG 51510	Ethiopia	Landraces
IFPI 2352	ICARDA-Siria	*P. sativum* subsp. *sativum* var. *arvense* (L.) Poir.	IG 51511	Ethiopia	Landraces
IFPI 2353	ICARDA-Siria	*P. sativum* subsp. *sativum* var. *arvense* (L.) Poir.	IG 51512	Ethiopia	Landraces
IFPI 2354	ICARDA-Siria	*P. sativum* subsp. *sativum* var. *arvense* (L.) Poir.	IG 51513	Ethiopia	Landraces
IFPI 2356	ICARDA-Siria	*P. sativum* subsp. *sativum* var. *arvense* (L.) Poir.	IG 51515	Ethiopia	Landraces
IFPI 2357	ICARDA-Siria	*P. sativum* subsp. *sativum* var. *arvense* (L.) Poir.	IG 51516	Ethiopia	Landraces
IFPI 2358	ICARDA-Siria	*P. sativum* subsp. *sativum* var. *arvense* (L.) Poir.	IG 51517	Ethiopia	Landraces
IFPI 2360	ICARDA-Siria	*P. sativum* subsp. *sativum* var. *arvense* (L.) Poir.	IG 51519	Ethiopia	Landraces
IFPI 2362	ICARDA-Siria	*P. sativum* subsp. *sativum* var. *arvense* (L.) Poir.	IG 51521	Ethiopia	Landraces
IFPI 2363	ICARDA-Siria	*P. sativum* subsp. *sativum* var. *arvense* (L.) Poir.	IG 51522	Ethiopia	Landraces
IFPI 2364	ICARDA-Siria	*P. sativum* subsp. *sativum* var. *arvense* (L.) Poir.	IG 51523	Ethiopia	Landraces
IFPI 2365	ICARDA-Siria	*P. sativum* subsp. *sativum* var. *arvense* (L.) Poir.	IG 51524	Ethiopia	Landraces
IFPI 2367	ICARDA-Siria	*P. sativum* subsp. *sativum* var. *arvense* (L.) Poir.	IG 51526	Ethiopia	Landraces
IFPI 2369	ICARDA-Siria	*P. sativum* subsp. *sativum* var. *arvense* (L.) Poir.	IG 51528	Ethiopia	Landraces
IFPI 2370	ICARDA-Siria	*P. sativum* subsp. *sativum* var. *arvense* (L.) Poir.	IG 51529	Ethiopia	Landraces
IFPI 2371	ICARDA-Siria	*P. sativum* subsp. *sativum* var. *arvense* (L.) Poir.	IG 51530	Ethiopia	Landraces
IFPI 2372	ICARDA-Siria	*P. sativum* subsp. *sativum* var. *arvense* (L.) Poir.	IG 51531	Ethiopia	Landraces
IFPI 2441	ICARDA-Siria	*P. sativum* subsp. *jomardii* (Schrank) Kosterin	IG 51600	Denmark	Unknown
IFPI 2495	ICARDA-Siria	*P. sativum* subsp. *jomardii* (Schrank) Kosterin	IG 51654	UK	Landraces
IFPI 3232	ICARDA-Siria	*P. fulvum* Sm.	IG 52391	Syria	Wild
IFPI 3250	ICARDA-Siria	*P. sativum* L.	IG 52409	Syria	wild
IFPI 3252	ICARDA-Siria	*P. sativum* subsp. *elatius* (M. Bieb.) Asch. & Graebn.	IG 52411	Syria	wild
IFPI 3253	ICARDA-Siria	*P. fulvum* Sm.	IG 52412	Syria	wild
IFPI 3257	ICARDA-Siria	*P. fulvum* Sm.	IG 52416	Syria	wild
IFPI 3260	ICARDA-Siria	*P. fulvum* Sm.	IG 52419	Syria	wild
IFPI 3261	ICARDA-Siria	*P. fulvum* Sm.	IG 52420	Syria	wild
IFPI 3262	ICARDA-Siria	*P. fulvum* Sm.	IG 52421	Syria	wild
IFPI 3280	ICARDA-Siria	*P. sativum* subsp. *elatius* (M. Bieb.) Asch. & Graebn.	IG 52439	Syria	wild
IFPI 3282	ICARDA-Siria	*P. sativum* subsp. *elatius* (M. Bieb.) Asch. & Graebn.	IG 52441	Syria	wild
IFPI 3330	ICARDA-Siria	*P. sativum* subsp. *elatius* var. *elatius* (M. Bieb.) Alef.	IG 52489	Turkey	wild
IFPI 3334	ICARDA-Siria	*P. sativum* subsp. *elatius* var. *elatius* (M. Bieb.) Alef.	IG 52493	Turkey	wild
IFPI 3338	ICARDA-Siria	*P. sativum* subsp. *elatius* var. *elatius* (M. Bieb.) Alef.	IG 52497	Turkey	wild
IFPI 3358	ICARDA-Siria	*P. sativum* subsp. *elatius* var. *elatius* (M. Bieb.) Alef.	IG 52517	Turkey	wild

	IAS - CSIC	*P. sativum* subsp. *elatius* var. *elatius* (M. Bieb.) Alef.		Unknow	wild
JI 1006	JIC	*P. fulvum* Sm.	WBH 2142	Israel	wild

Supplementary Table S2. Differences between groups in pea panel grouped by material type, growth condition and taxonomy.

Grouped by[a]	Group 1	Group 2	Signif.[b]
Material type	Breeding Line	Cultivar	*
Material type	Cultivar	Landraces	****
Material type	Cultivar	Wild	****
Growing Condition	DS_{2018}	DS_{2019}	***
Growing Condition	DS_{2018}	DS_{2020}	****
Growing Condition	DS_{2019}	DS_{2020}	****
Growing Condition	DS_{2018}	DS_{CC}	****
Growing Condition	DS_{2019}	DS_{CC}	****
Growing Condition	DS_{2020}	DS_{CC}	****
Taxonomy	*P. fulvum* Sm.	*P. sativum* subsp. *sativum* var. sativum	**
Taxonomy	*P. sativum* L.	*P. sativum* subsp. *sativum* var. sativum	****
Taxonomy	*P. sativum* subsp. *sativum* var. arvense	*P. sativum* subsp. *sativum* var. sativum	**
Taxonomy	*P. sativum* subsp. *sativum* var. sativum	*P. sativum* subsp. *elatius* var. elatius	****

[a] Grouped by column attend to Material type (Breeding Line, Cultivar, Landrace, Wild or Unknown), Growth Condition (Disease severity in Controlled Conditions (DS_{CC}) or Disease Severity in environments Co-2018, Co-2019 or Co-202 (DS_{2018}, DS_{2019} and DS_{2020}, respectively)) and *Pisum* taxonomy.

[b] Signif. column reveals the statistical significance in the Wilcoxon test: * = $p < 0,05$, ** = $p < 0,01$, *** = $p < 0,001$, **** = $p < 0,0001$
